# Preconditioning in hypoxic-ischemic neonate mice triggers Na^+^-Ca^2+^ exchanger-dependent neurogenesis

**DOI:** 10.1038/s41420-022-01089-z

**Published:** 2022-07-13

**Authors:** P. Brancaccio, S. Anzilotti, O. Cuomo, A. Vinciguerra, M. Campanile, A. Herchuelz, S. Amoroso, L. Annunziato, G. Pignataro

**Affiliations:** 1 Division of Pharmacology, Department of Neuroscience, School of Medicine, University of Naples “Federico II”, 80131 Naples, Italy; 2 Department of Science and Technology, University of Sannio, 82100 Benevento, Italy; 3 Department of Biomedical Sciences and Public Health, School of Medicine, University “Politecnica delle Marche”, 60126 Ancona, Italy; 4 Laboratoire de Pharmacodynamie et de Therapeutique—Faculté de Médecine Université Libre de Bruxelles, Bruxelles, Belgium; 5 IRCCS Synlab SDN S.p.A, via Gianturco 113, 80143 Naples, Italy

**Keywords:** Preclinical research, Cell death in the nervous system

## Abstract

To identify alternative interventions in neonatal hypoxic-ischemic encephalopathy, researchers’ attention has been focused to the study of endogenous neuroprotective strategies. Based on the preconditioning concept that a subthreshold insult may protect from a subsequent harmful event, we aimed at identifying a new preconditioning protocol able to enhance Ca^2+^-dependent neurogenesis in a mouse model of neonatal hypoxia ischemia (HI). To this purpose, we also investigated the role of the preconditioning-linked protein controlling ionic homeostasis, Na^+^/Ca^2+^ exchanger (NCX). Hypoxic Preconditioning (HPC) was reproduced by exposing P7 mice to 20’ hypoxia. HI was induced by isolating and cutting the right common carotid artery. A significant reduction in ischemic damage was observed in mice subjected to 20’ hypoxia followed,3 days later, by 60’ HI, thus suggesting that 20’ hypoxia functions as preconditioning stimulus. HPC promoted neuroblasts proliferation in the dentate gyrus mirrored by an increase of NCX1 and NCX3-positive cells and an improvement of behavioral motor performances in HI mice. An attenuation of HPC neuroprotection as well as a reduction in the expression of neurogenesis markers, including p57 and NeuroD1, was observed in preconditioned mice lacking NCX1 or NCX3. In summary, PC in neonatal mice triggers a neurogenic process linked to ionic homeostasis maintenance, regulated by NCX1 and NCX3.

## Introduction

Neonatal hypoxic-ischemic encephalopathy (HIE) is a major cause of acute mortality and chronic neurologic deficit in infants and children. It is caused by a reduction of cerebral blood flow (CBF) in the brain [1, 2], that in turn is due to the alteration of respiratory placental or pulmonary gas exchange [3–5]. Neonatal HIE represents a medical need since therapeutic hypothermia represents the only accepted treatment so far available [6]. In order to identify new druggable targets and to settled up innovative therapeutic approaches, the attention of researchers has been recently focused to the study of endogenous neuroprotective strategies, including ischemic preconditioning (PC), a strategy able to protect the brain and other organs by subjecting them to a subthreshold injurious stimulus before a longer harmful ischemia [7–9]. Since during stressful and pathological conditions brain may activate neurogenetic program able to preserve its integrity [10, 11], the induction of neurogenesis as a potential neurorestorative strategy has been included among the possible mechanisms of neuroprotection elicited by preconditioning. Neurogenesis is the process by which new neurons are generated from progenitor cells located in specific areas of the brain, i.e., hippocampal dentate gyrus (DG) and subventricular zone (SVZ), called neurogenic niches, characterized by specialized microenvironments that support their self-renewal and differentiation throughout their life [12]. This process is highly complex and is finely regulated by numerous molecular pathways, such as the transcription factor NeuroD1 that plays a crucial role in the neuronal differentiation process [13–15], both during embryonic neurogenesis [16] and adult neurogenesis [17]. Notably, NeuroD1 is able to induce Ca^2+^-dependent neuronal differentiation [18, 19], thus supporting a Ca^2+^-dependent neurogenesis pathway.

In the light of these considerations, it is conceivable that the regulation of ionic homeostasis plays a fundamental role in promoting regenerative processes. Indeed, cell death during cerebral ischemia is characterized by the progressive intracellular accumulation of [Na^+^] and [Ca^2+^] ions. Maintaining the homeostasis of these two ions is crucial for proper cellular functioning. In fact, the plasmamembrane transporter Na^+^/Ca^2+^ exchanger (NCX), by contributing to the maintenance of ionic homeostasis, represents a key protein in controlling the progression of ischemia-induced brain damage [20, 21].

Considering these premises, in the present work we settled a new model of neonatal mouse hypoxic preconditioning in order to: (1) examine the proliferation and differentiation of neural stem cells (NSCs) in ipsilateral DG;(2) investigate the relationship between the endogenous neurogenesis, promoted by preconditioning, andNCX1 and NCX3, two isoforms of the NCX antiporter involved in stroke neuroprotection.

## Results

### Radial glial-like cells, monitored by Nestin, increase in the dentate gyrus 24 h after 20’ hypoxia in P8 mice

**Fig. 1:**
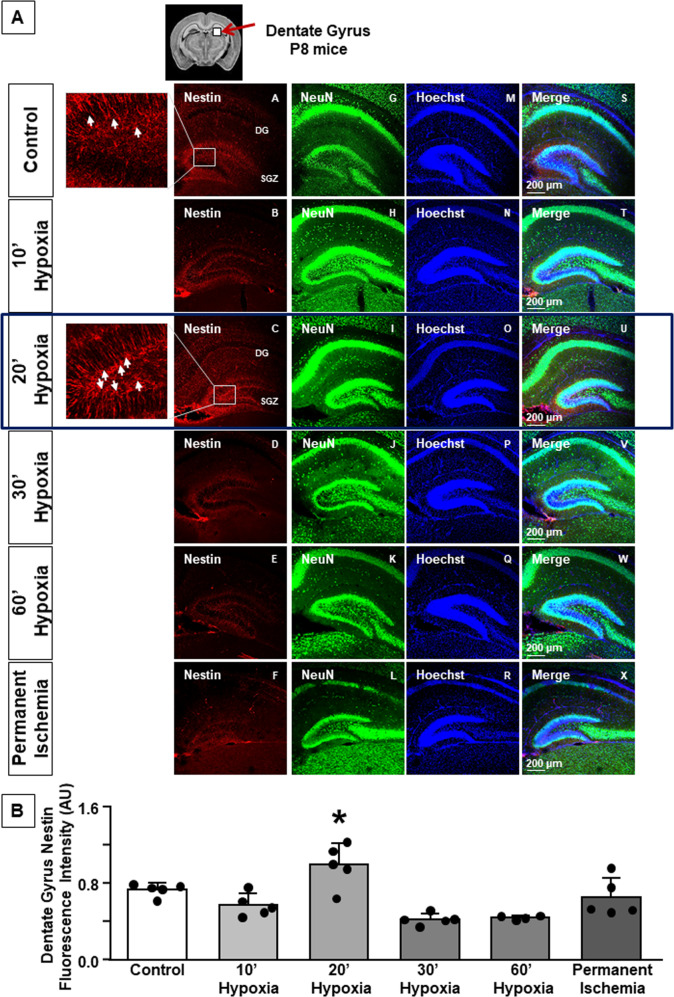
Nestin fluorescence intensity in dentate gyrus of P8 mice. **A** Representative confocal images of DG of control, 10’ hypoxia, 20’ hypoxia, 30’ hypoxia, 60’ hypoxia, permanent ischemia groups. Single staining of Nestin (A, B, C, D, E, F); NeuN (G, H, I, J, K, L), Hoechst (M, N, O, P, Q, R), and merge (S, T, U, V, W, X) in mouse dentate gyrus of six groups. Scale bar 200 μm. Rectangular box indicates area of high magnification ×400 zoom. Arrows indicate radial glial-like Nestin expressing cells. **B** Quantification of Nestin fluorescence intensity in dentate gyrus (expressed in arbitrary units, AU). Data are expressed as mean ± SD (*n* = 4–5). *P < 0.05 versus control mice. *P*-values were obtained by using one-way ANOVA with Newman–Keuls correction for multiple comparisons.

To analyze the expression of radial glial-like cells in DG niche, Nestin fluorescence intensity was assessed. Nestin antibody allowed to identify an intermediate filament protein type VI (IFN), a component of neural progenitor cytoskeleton [22], that significantly increases during differentiation of stem cells [23, 24]. A significant increase in Nestin expression was observed in mice subjected to 20’ hypoxia. In addition, a significant reduction in Nestin was observed in mice subjected to 30’ and 60’ hypoxia compared both to control and 20’ hypoxia groups (*P* < 0.05) (Fig. 1A, B). No significant changes were observed in mice subjected to permanent ischemia (Fig. 1A, B). As expected, Nestin expression was absent in gastrocnemius muscle (Fig. S1).

### 20’ hypoxia reduces ischemic and hippocampal damage in hypoxic-ischemic neonatal mice and functions as preconditioning stimulus

**Fig. 2:**
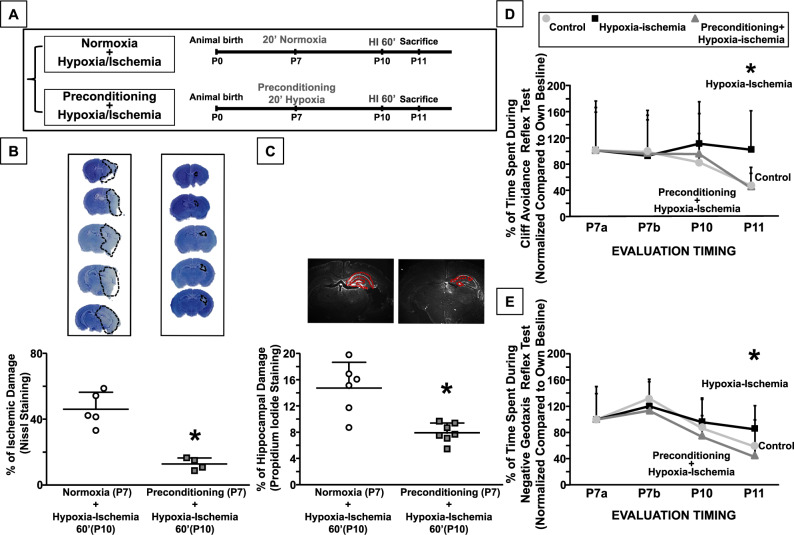
Effect of 20’ hypoxia in hypoxic-ischemic neonatal mice. **A** The panel shows the experimental design. The mice were divided into two groups: the first group was subjected to normoxic condition at P7; in the second the mice were subjected to 20’ hypoxia at P7, subsequently, all animals were subjected to HI insult at P10. All animals were sacrificed 24 h after injury. **B** On top of the figure are included representative rostral-caudal brain sections stained with cresyl violet. Brain damage was expressed as % of ischemic damage 24 h after HI induction. Data are expressed as mean ± SD (*n* = 4–5). **C** On top of the figure are included representative brain sections stained with PI. Data are expressed as mean ± SD (*n* = 6–7). **B**, **C** *P < 0.05 versus mice group subjected to only HI (P10). *P*-values were obtained by using Unpaired *t*-test. **D**, **E** The graphs show the time course of cliff avoidance and negative geotaxis reflex performances into three experimental groups. Mice were monitored at different time intervals. Data are expressed as mean ± SD (*n* = 15–16). *P < 0.05 versus control mice and preconditioned hypoxic-ischemic mice. *P*-values were obtained by using one-way ANOVA with Newman–Keuls correction for multiple comparisons.

Ischemic damage was evaluated by cresyl violet staining in P7 mice subjected to 20’ hypoxia followed, at P10, by ischemia plus 60’ hypoxia (HI) and sacrificed 24 h after the lethal insult. Interestingly, 20’ hypoxia worked as preconditioning stimulus (HPC), thus reducing ischemic damage (*P* < 0.05) (Fig. 2B). The effect of HPC was evident also when brain damage was assessed with PI technique [25, 26] (Fig. 2C). The development of sensorimotor reflexes was evaluated by cliff avoidance and negative geotaxis tests. P11 preconditioned hypoxic-ischemic mice spent less time to perform the trials compared to hypoxic-ischemic mice (*P* < 0.05) (Fig. 2D, E).

### HPC prevents the reduction of total number of proliferating neuroblasts, monitored by PSA-NCAM, in dentate gyrus of hypoxic-ischemic mice

**Fig. 3:**
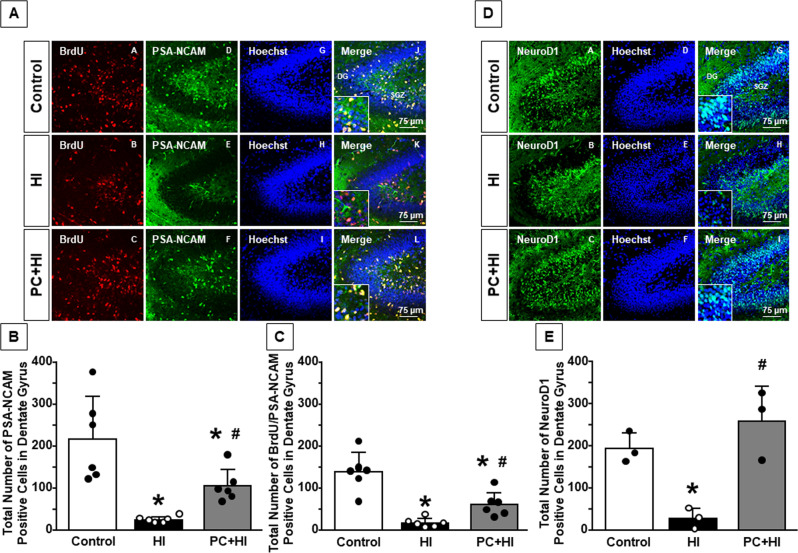
Effect of hypoxic preconditioning on proliferating neuroblasts in dentate gyrus of HI mice. **A** Representative confocal images of dentate gyrus of control, HI, and PC + HI groups. Single staining of BrdU (A, B, C), PSA-NCAM (D, E, F), Hoechst (G, H, I), and merge (J, K, L). Scale bar 75 μm. Rectangular box indicates area of high magnification ×500 zoom. **B** Cell-counting analysis of neuroblasts expressed as the total number of PSA-NCAM^+^ cells in DG of P11 mice. **C** Cell-counting analysis of proliferating neuroblasts expressed as the total number of BrdU^+^/PSA-NCAM^+^ cells in DG of P11 mice. **B**, **C** Data are expressed as mean ± SD (*n* = 6). *P < 0.05 versus control mice. ^#^*P* < 0.05 versus mice group subjected to only HI. *P*-values were obtained by using one-way ANOVA with Newman–Keuls correction for multiple comparisons. **D** Representative confocal images of DG of control, HI, and PC + HI groups. Single staining of NeuroD1 (A, B, C), Hoechst (D, E, F), and merge (G, H, I). Scale bar 75 μm. Rectangular box indicates area of high magnification ×500 zoom. **E** Cell-counting analysis of immature neurons expressed as total number of NeuroD1^+^cells in DG of P11 mice. Data are expressed as mean ± SD (*n* = 3). *P < 0.05 versus control mice. ^#^*P* < 0.05 versus mice group subjected to only HI. *P*-values were obtained by using one-way ANOVA with Newman–Keuls correction for multiple comparisons.

Proliferating neuroblasts were evaluated by anti-BrdU and anti-PSA-NCAM antibodies. BrdU is incorporated in dividing progenitor cells and passed on to their daughter cells [27] thus identifying proliferating cells, while PSA-NCAM antibody identifies a membrane-bound glycoprotein expressed by neuroblasts at their surface [22]. PSA-NCAM staining in DG showed that mice exposed to HI displayed a reduction in neuroblast number compared to control animals, while mice exposed to PC + HI displayed an increase in the number of PSA-NCAM-positive cells compared to HI mice (*P* < 0.05) (Fig. 3A, B); this effect was also revealed in SVZ (*P* < 0.05) (Fig. S2A, B). Interestingly, mice subjected to PC + HI showed an increased number of proliferating neuroblasts in DG compared to mice subjected to HI alone (*P* < 0.05) (Fig. 3A–C).

PC significantly prevented the reduction of neurogenic differentiation factor NeuroD1, expressed from immature neurons, in the DG of HI mice (*P* < 0.05) (Fig. 3D, E).

To verify if preconditioning alone was able to enhance the increase of neuronal progenitors in DG, Nestin expression was analyzed in four experimental groups: control, PC, HI, and PC + HI mice. 20’ hypoxia was not able to determine an increase of Nestin in P11 mice, conversely preconditioning stimulus followed by hypoxic-ischemic insult induced a rise of Nestin-positive cells compared to HI group (Fig. S3).

### Preconditioning prevented NCX1 and NCX3 reduction induced by hypoxia ischemia in the dentate gyrus of P11 mice

**Fig. 4:**
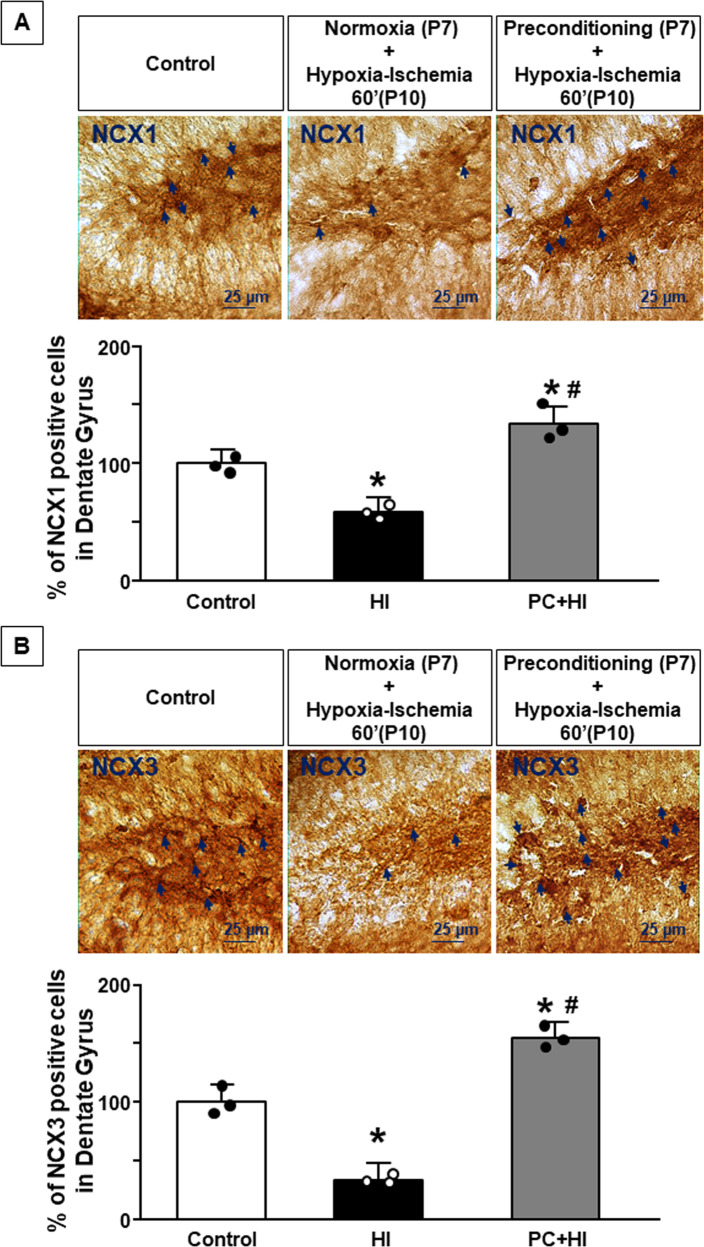
NCX1 and NCX3 expression in the dentate gyrus after HPC. **A** Representative image of NCX1 3,3′-diaminobenzidine (DAB) staining of dentate gyrus of control, HI, and PC + HI groups. Scale bar 25 μm. Arrows indicate NCX1 expressing cells. Cell-counting analysis was expressed as the percentage of total number of NCX1^+^cells in dentate gyrus of P11 mice. **B** Representative image of NCX3 DAB staining of dentate gyrus of control, HI, and PC + HI groups. Scale bar 25 μm. Arrows indicate NCX3 expressing cells. Cell-counting analysis was expressed as the percentage of total number of NCX3^+^ cells in dentate gyrus of P11 mice. **A**, **B** Data are expressed as mean ± SD (*n* = 3). *P < 0.05 versus control mice. ^#^*P* < 0.05 versus mice group subjected to HI only. *P*-values were obtained by using one-way ANOVA with Newman–Keuls correction for multiple comparisons.

To verify whether NCX1 and NCX3 were involved in preconditioning-induced neuroprotection, their expression was evaluated in ipsilateral DG of three experimental groups: control, hypoxic-ischemic, and preconditioned hypoxic-ischemic mice. The counting analysis of NCX1 and NCX3 immunostaining revealed a significant increase in NCX1 and NCX3-positive cells in DG of mice subjected to PC + HI compared to both control and HI mice (*P* < 0.05) (Fig. 4A, B).

### NCX1 silencing and ncx3 genetic knocked-out worsen infarct volume and neurological deficits in HI mice and impair preconditioning-induced neuroprotection

**Fig. 5:**
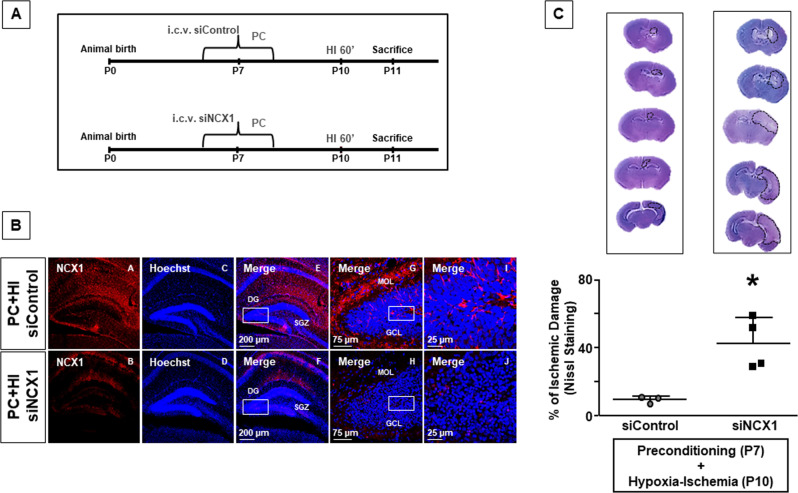
Effect of NCX1 silencing on preconditioning-induced neuroprotection. **A** The panel shows the experimental design. The mice were divided into two groups: the first group was subjected to i.c.v. administration of siControl and HPC at P7; in the second the mice were subjected to i.c.v. administration of siNCX1 and HPC at P7, subsequently, all animals were subjected to HI insult at P10. All animals were sacrificed 24 h after injury. **B** Representative confocal images of hippocampus of two groups. Single staining of NCX1 (A, B); Hoechst (C, D), and merge (E, F, G, H, I, J) in hippocampus of two groups. Scale bar: 200 μm (E, F), 75 μm (G, H), 25 μm (I, J). Rectangular box indicates area of higher magnification. MOL, molecular layer; GCL, granular cell layer. **C** On top of the figure are included representative rostral-caudal brain sections stained with cresyl violet of preconditioned hypoxic-ischemic (siControl) and preconditioned hypoxic-ischemic (siNCX1) mice. Brain damage induced in mice was evaluated as % of ischemic damage at postnatal day eleven. Data are expressed as mean ± SD (*n* = 3–4). *P < 0.05 versus preconditioned hypoxic-ischemic (siControl) mice. *P*-values were obtained by using Unpaired *t*-test.

siNCX1 intracerebroventricular injected in mice exposed to HI was able to reduce the expression of NCX1 in the hippocampal DG (Fig. 5B) and to prevent the neuroprotection mediated by HPC (*P* < 0.05) (Fig. 5C). In particular, the reduction of NCX1 expression caused the increase in necrotic and apoptotic cells in granular cell layer (Fig. 5B–H); high-magnification image (Fig. 5B–J) showed pycnotic nuclei, characterized by karyolysis and karyorrhexis.

**Fig. 6:**
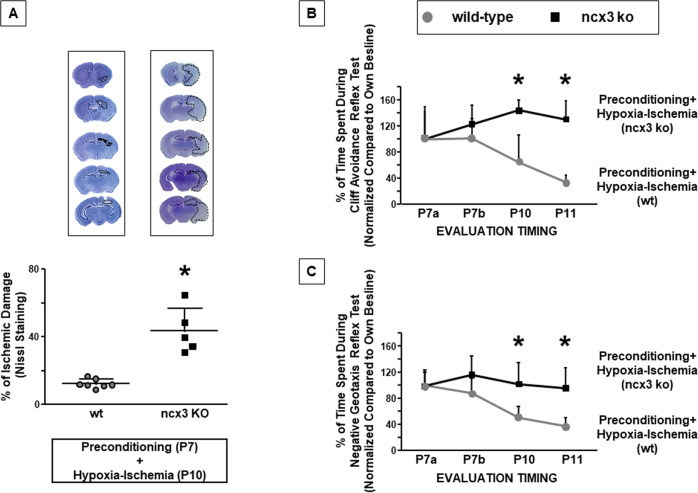
Effect of ncx3 genetic knocked-out on preconditioning-induced neuroprotection. **A** On top of the figure are included representative rostral-caudal brain sections stained with cresyl violet of preconditioned hypoxic-ischemic (wild-type) and preconditioned hypoxic-ischemic (ncx3−/−) mice. Brain damage induced in mice was evaluated as % of ischemic damage at P11. Data are expressed as mean ± SD (*n* = 5–7). *P < 0.05 versus preconditioned hypoxic-ischemic (wild-type) mice. *P*-values were obtained by using Unpaired *t*-test. **B**, **C** The graphs show the time course of cliff avoidance and negative geotaxis reflex performances into two experimental groups: preconditioned hypoxic-ischemic (wild-type) and preconditioned hypoxic-ischemic (ncx3−/−) mice. Mice were monitored at different time intervals. Data are expressed as mean ± SD (*n* = 7–8). *P < 0.05 versus PC + HI mice (wild-type). *P*-values were obtained by using Unpaired *t*-test.

ncx3 genetic ablation prevented the neuroprotection induced by hypoxic preconditioning in mice ipsilateral brain (*P* < 0.05) (Fig. 6A).

ncx3−/− mice subjected to PC + HI spent more time to perform the trials in cliff avoidance and negative geotaxis tests, compared to wild-type mice (*P* < 0.05) (Fig. 6B, C). PC protective effect on unconditional sensory-motor reflexes was absent in preconditioned hypoxic-ischemic ncx3−/− mice.

### NCX1 silencing and ncx3 genetic knocked-out prevented the preconditioning-induced differentiation phase, monitored by NeuroD1

To examine whether the role of NCX1 and NCX3 was related to the neurogenesis process elicited by PC in DG of hypoxic-ischemic mice, the expression of p57, an important regulator of the cell cycle, a marker of radial quiescent NSCs [28] and NeuroD1, a marker of neuroblast differentiation, was evaluated in mice in which ncx1 was silenced or ncx3 was knocked-out.

**Fig. 7:**
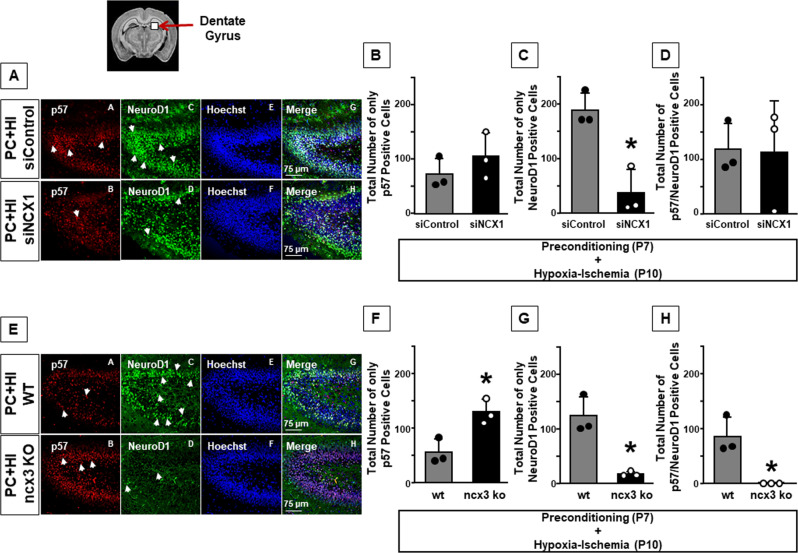
Effect of NCX1 silencing and ncx3 genetic knocked-out on the differentiation induced by HPC. **A** Representative confocal images of dentate gyrus of preconditioned hypoxic-ischemic mice (siControl) and preconditioned hypoxic-ischemic mice (siNCX1). Single staining of p57 (A, B), NeuroD1 (C, D), Hoechst (E, F), and merge (G, H). Scale bar 75 μm. Arrows indicate p57 and NeuroD1 expressing cells. **B** Cell-counting analysis of total number of onlyp57^+^ cells in DG of P11 mice. **C** Cell-counting analysis of total number of only NeuroD1^+^ cells in DG of P11 mice. **D** Cell-counting analysis of total number of p57^+^/NeuroD1^+^ cells in DG of P11 mice. **B**, **C**, **D** Data are expressed as mean ± SD (*n* = 3). **C** *P < 0.05 versus preconditioned hypoxic-ischemic mice (siControl). *P*-values were obtained by using Unpaired *t*-test. **E** Representative confocal images of dentate gyrus of preconditioned hypoxic-ischemic (wild-type) and preconditioned hypoxic-ischemic (ncx3−/−) groups. Single staining of p57 (A, B), NeuroD1 (C, D), Hoechst (E, F), and merge (G, H). Scale bar 75 μm. Arrows indicate p57 and NeuroD1 expressing cells. **F** Cell-counting analysis of total number of only p57^+^cells in DG of P11 mice. **G** Cell-counting analysis of total number of only NeuroD1^+^ cells in DG of P11 mice. **H** Cell-counting analysis of total number of p57^+^/NeuroD1^+^ cells in DG of P11 mice. **F**, **G**, **H** Data are expressed as mean ± SD (*n* = 3). *P < 0.05 versus preconditioned hypoxic-ischemic mice (wild-type). *P*-values were obtained by using Unpaired *t*-test.

Silencing of NCX1 or genetic ablation of ncx3 prevented the increase of NeuroD1-positive cells elicited by hypoxic preconditioning, thus suggesting a role of these two NCX isoforms in the differentiation phase of neurogenesis (*P* < 0.05) (Fig. 7).

Notably, no effect on p57 expression was found in DG of preconditioned mice while the lack of ncx3 induced a strong increase in p57 expression in the same brain region (Fig. 7E, F).

In the DG of ncx3 KO mice exposed to PC alone the number of NeuroD1 expressing cells at postnatal day eleven was reduced compared to wild-type animals (Fig. S4). The reduction of NeuroD1 expressing cells was also observed in hypoxic-ischemic mice, where the strong insult determined the increase of apoptotic cells in granular cell layer. Interestingly, in hypoxic-ischemic ncx3 KO mice a deep alteration of DG morphology was observed (Fig. S5).

## Discussion

In the present paper, we demonstrated, for the first time that a subthreshold hypoxic event of 20 min, that does not induce brain damage, represents a reliable and valid preconditioning stimulus in a mouse model of neonatal hypoxia ischemia. In fact, this hypoxic preconditioning stimulus is associated with endogenous neurogenesis in immature brain. Furthermore, we demonstrated that the increase of neurogenic processes triggered by HPC may be linked to ionic perturbation, since NCX1 and NCX3, two isoforms of the antiporter sodium/calcium exchanger, that play a relevant role in the maintenance of intracellular sodium and calcium homeostasis, were upregulated by HPC. Indeed, NCX1 silencing or NCX3 knocking-out, prevented HPC-induced neurogenesis, mainly in the differentiation stage.

This work further supports the idea that preconditioning represents a general paradigm applicable to different species and pathological conditions. In fact, moderate hypoxic episodes may exert beneficial actions, as illustrated by preconditioning experiments carried out mainly in adult rats where non-lethal hypoxia induces brain tolerance to subsequent severe insults [29, 30], a process, which may involve neurogenesis enhancement [31–34].

Interestingly, in 2007, Yin et al. demonstrated, in a rat model of neonatal hypoxia ischemia, that the exposure for 3 h to a humidified gas mixture of 8% oxygen 92% nitrogen induced a state of tolerance, thus determining a significant reduction of tissue loss 24 h and 7 days after HI induction [35]. Differently from the 3 h hypoxia previously used as preconditioning stimulus in neonate rats [35], here we demonstrated that a more easily applicable 20’ hypoxia is able to work as preconditioning stimulus in P7-P10 mice. The choice of carrying out these studies in animals of this age was made with the intention to replicate a mouse model as closely as possible similar to the human neonates affected by hypoxic-ischemic encephalopathy at birth. In fact, it is known that brain development at P10 in mice corresponds to the brain development of a human at birth [36, 37]. Interestingly, it has been demonstrated that the time interval between P7-P14, is characterized by the strongest increase in proliferating progenitor cells in the DG of mouse hippocampus [38].

An important aspect of our study that deserves to be underlined concerns the relationship between the maintenance of ionic homeostasis mediated by NCX and the activation of endogenous neurogenesis. In fact, the silencing of ncx1, as well as the genetically induced ablation of ncx3, prevent neurogenesis induced by preconditioning in P10 mice. Unfortunately, ncx1 knock-out mice are not vital [39], therefore we choose to silence this NCX1 isoform to dissect its role. These findings agree with a proposed role of NCX in neuronal differentiation. Indeed, we have previously demonstrated in vitro that the overexpression of NCX1 was correlated with neuronal differentiation and neurite outgrowth, occurring though Akt phosphorylation [40, 41]. In this regard, it is notable to mention that Yin et al. reported that PI3K/Akt pathway is activated by preconditioning in neonatal rats [35], thus further supporting the relationship between NCX and PI3K/AKT pathway also in neonatal preconditioning.

Notably, we have previously demonstrated that NCX1 and NCX3 represent two new molecular effectors involved in adult brain preconditioning [20, 42, 43]. Indeed, the lack of NCX1 and NCX3 induced by their respective silencing prevented ischemic preconditioning neuroprotection in adult rats [20, 42]. Here, we demonstrated that a pronounced increase of NCX1 and NCX3-positive cells within DG of preconditioned hypoxic-ischemic mice occurred. We hypothesize that this event occurs to counteract the dysregulation of intracellular Na^+^ and Ca^2+^ homeostasis triggered by harmful ischemia. The pivotal role played by NCX1 and NCX3 isoforms during preconditioning has been further highlighted also in in vitro studies. In fact, in rat primary cortical neurons IPC induced an increase in NCX1 and NCX3 isoform expression and this effect was mediated by nitric oxide (NO) activation of PI3K/Akt pathway [43].

Since the effect of preconditioning induced in mice lacking NCX1 or NCX3 was more evident on the transcription factor NeuroD1 [17], mainly involved in the differentiation stage of NSCs, the impact of NCX1 and NCX3 on the neurogenetic process elicited by preconditioning is likely directed to the differentiation stage.

In conclusion, our results indicate that hypoxic preconditioning is associated with endogenous neurogenesis in immature brain. Indeed, this stimulus triggers the proliferation of neural progenitor cells in the neurogenic niches. The increase of neurogenic processes might be related to ionic homeostasis maintenance, regulated by NCX1 and NCX3 isoforms of the antiporter sodium/calcium exchanger. Therefore, NCX1 and NCX3 might represent potential druggable targets for brain damage associated with neonatal hypoxic-ischemic encephalopathy.

## Material and methods

### Animal procedures

Two-hundred and one (201) male and female mice, C57BL/6 (Charles River, Italy), housed under diurnal lighting conditions (12 h darkness/light) were used, 41 out of 201 animals were not included in the experimental groups for the following reasons: 11 were not cured by their dams; 30 died immediately after surgery. G*Power version 3.1.9.7 was used to determine sample size. NCX3 knock-out mice were generated by our research group as previously described [44]. Genetic background of ncx3+/− and ncx3−/− mice were obtained as previously described [45]. All animal procedures were performed in accordance with Animal Care Committee of “Federico II” University of Naples, Italian Ministry Authorization (DL26/2014). All protocols and procedures were in accordance with the international guiding principles for biomedical research proposed by the Council for International Organizations of Medical Sciences and ARRIVE (Animal Research: Reporting In Vivo Experiments) guidelines [46].

### Hypoxic-ischemic surgical procedure

Hypoxic-ischemic injury was induced in P10 mice according to Rice-Vannucci model [21, 47, 48], with few modifications. In particular, mice were anesthetized with 1.5% sevoflurane, and 98.5% O_2_ (Oxygen concentrator, Longfei Industry Co, Zhejiang, China; Mod. LFY-I-5). The body temperature was maintained at 37 ± 0.5 °C during the whole procedure with a heating pad, and under a surgical stereomicroscope, a midline skin incision (0.5 cm) was made on the neck, and the right common carotid artery (CCA) was exposed and cut. Animals were returned to their dams and monitored continuously during a recovery period of 1 h. After that, pups were subjected to 60’ hypoxia (8% O_2_, 92% N_2_) in a hypoxic chamber at 37 °C (GOX 100T, Greisinger, Germany). At the end of the procedure, pups were returned to their dams. Animals were monitored daily until they were sacrificed [21].

### Hypoxic preconditioning

P7 mice were subjected to hypoxic preconditioning induced by 20 min of hypoxia, into a hypoxic chamber, perfused with an equilibrated gaseous mixture (8% O_2_ and 92% N_2_) monitored by using an oxygen monitor. The hypoxic chamber was placed in a water bath heated to 37 °C. Control pups not subjected to hypoxic preconditioning were exposed to room air in a chamber for the same time intervals. At the end of the procedure, all pups were returned to their dams. Pups were then subjected to HI and sacrificed 24 h after insult induction.

### siNCX1 administration

Neonatal mice were anesthetized with 1.5% sevoflurane and 98.5% O_2_ gaseous mixture. Their heads were immobilized on a stereotaxic apparatus and a midline sagittal skin incision (0.5 cm) was made on the skull. Scramble silencing or siNCX1 (0,5 µL, 5 µM/L) (GenBank NM_019268) (Qiagen, Milan, Italy; SI03085285) were injected at the following stereotaxic coordinates identified from the bregma: 0 mm antero-posterior, 0.8 mm lateral, and 1.5 mm depth [49], with a Hamilton micro-syringe, 8 h before preconditioning induction. Pups were then placed on a heat pad, quickly revitalized, and returned to their dams.

### BrdU administration

BrdU (100 mg/kg body weight, 10 mg/mL in 0.007 N NaOH in 0.9% NaCl) (Abcam, Cambridge, UK; ab142567) was ip administered at postnatal day 7 [27]. Pups were then exposed to hypoxic preconditioning or normoxic conditions. At postnatal day 11, mice were transcardially perfused and brain tissue processed as described below.

### Brain damage analysis

To evaluate hippocampal damage, 2 µL of Propidium Iodide solution (PI, 1 mg/mL in distilled water, Sigma, Milan, Italy; P4170) was injected into the right lateral brain ventricle of HI newborn mice, as previously described [21]. Brains were rapidly removed on ice and postfixed overnight at +4 °C and cryoprotected in 30% sucrose in 0.1 M phosphate buffer (PB) with 0.02% sodium azide for 24 h at +4 °C. Brains were cut on a cryostat into 100 µm coronal sections in rostral-caudal direction. Brain damage was then assessed with propidium iodide technique [25, 26]. Consecutive sections were analyzed with fluorescence microscope (Nikon E400) and the PI-stained infarct area was calculated with image analysis software (Image-J) [50]. To avoid edema interference in damage quantification, hippocampal damage was expressed as percentage of the ratio between the sum of the infarct in hippocampal areas and the sum of the total areas of the respective ipsilateral hippocampus.

To determine total brain damage, brains were removed and cut into 40 µm coronal sections in rostral-caudal direction. The evaluation of brain damage was carried out by Nissl staining [21], to avoid edema interference in damage quantification, ischemic damage was expressed as percentage of the ratio between ischemic damage and total area of the ipsilateral hemisphere.

### Tissue processing, immunostaining, and confocal immunofluorescence

Immunostaining and confocal immunofluorescence procedures were performed as previously described [49, 51]. For BrdU staining, paraformaldehyde-fixed frozen sections were pretreated with 2 N HCl at RT for 30’ and washed three times in accordance with manufacturer’s protocol (Abcam, Cambridge, UK). Brain sections were incubated overnight at +4 °C with the following primary antibodies: anti-NeuN (1:1000, Millipore, Milan, Italy; ABN78); anti-Nestin (1:200, Millipore, Milan, Italy; MAB353) [52]; anti-alpha Tubulin 4a (1:400, GeneTex, Irvine, California, USA; GTX112141), anti-BrdU (1:300, Abcam, Cambridge, UK; ab6326); anti-PSA-NCAM (1:300, Millipore, Milan, Italy; MAB5324); anti-NeuroD1 (1:300, Abcam, Cambridge, UK;ab60704); anti-NCX1 (1:500, Swant, Bellinzona, Switzerland; R3F1), anti-p57 (1:500, Sigma, Milan, Italy; P0357). Sections were then incubated with the corresponding florescent-labeled secondary antibodies 1:300 (Alexa 488/Alexa 594 conjugated antimouse/antirabbit/antirat IgGs, 715-545-150, 711-585-152, Jackson Immuno Research, Baltimore, PA; and ab102262, Abcam, Cambridge, UK). Nuclei were counterstained with Hoechst (Sigma-Aldrich, Milan, Italy). Images were observed using a Zeiss LSM700 META/laser scanning confocal microscope (Zeiss, Oberkochen, Germany). Single images were taken with a resolution of 512 × 512. In double-labeled sections, the pattern of immune reactivity for both antigens was identical to that seen in single-stained material [53]. Nissl staining was performed as previously described [21]. For DAB staining, standard 3,3-diaminobenzidine (DAB) was employed on coronal sections using anti-NCX1 (1:500) and anti-NCX3 (1:3000) (Swant, Bellinzona, Switzerland; catalog 95,209) [54].

### Fluorescence intensity analysis

Nestin fluorescence was quantified in terms of pixel intensity on tissue sections at the level of the DG of hippocampus by using Image J software, as previously described [55, 56]. Images from the same areas of each brain region were compared, three selected rostro-caudal sections were obtained from the region 4.35 to 4.95 mm from rostral cortex [57] and included in the analysis. Mean values per sample were calculated by averaging the values of all sections of the same animal and were used to compare the Nestin fluorescence intensity of animals subjected to different stimuli vs. control animals.

### Cell-counting analysis

The area of the complete hilus, GCL, and subgranular zone of the DG, with an imaginary cut at the beginning of CA3, was manually counted in each section, as previously described [58].

The number of BrdU and PSA-NCAM-positive cells was determined in four matched sections (40 µm) of the medial DG of hippocampus of C57BL/6 mice at P11, by manual counting at ×40 magnification. The average value of all the sections of each animal was determined.

The number of NCX1 and NCX3-positive cells with clearly visible cell bodies and profiles was determined in four matched sections (40 µm) of the medial DG of hippocampus of C57BL/6 mice at P11, by manual counting at ×20 magnification [51].

The number of NeuroD1-positive cells was determined in three matched sections (40 µm) of the medial DG of hippocampus of C57BL/6 mice at P11.The number of NeuroD1 and p57-positive cells was determined in three and four matched sections (40 µm) of the medial DG of hippocampus of P11 mice in NCX1 silencing and NCX3 knocking-out experiments, respectively by manual counting at ×40 magnification. All immunostainings were blind quantified.

### Behavioral studies

Experiments were performed under diurnal lighting conditions between 03:00 p.m. and 06:00 p.m. *Cliff avoidance* and *Negative Geotaxis* tests were carried out at different time intervals. P7 mice were analyzed twice, 20 min before (P7a) and 20 min after normoxia o hypoxia (P7b). Afterwards, tests were replicated before HI induction (P10) and before the sacrifice (P11). Each pup was given three attempts with a maximum time of 30 s for each trial, the mean time to perform the reflex was recorded.

#### Cliff avoidance reflex test

Pups were located at the edge of a flat surface, such that the forepaws and snout of the pups are over the edge. The time spent to move away by backing up with forepaws or to retract their body and to turn head sideways was calculated [59, 60]. If the mouse was unable to perform the test within the allotted time (30”) or fell off from the flat surface, the maximal time was assigned.

#### Negative geotaxis reflex test

Pups were placed head downward on an inclined board (40°) [60]. It has been considered the time required for the pups to rotate their bodies head up (>90° rotation).

### Statistical analysis

Statistical analyses were performed by using GraphPad Prism 5.0 (Graph Pad Software, Inc.). All data in figures represent the mean ± SD. Statistical differences between two experimental groups were analyzed with unpaired two tailed *t*-test. Statistical differences between more than two experimental groups were evaluated by one-way ANOVA, followed by Newman–Keuls test. Statistical significance was accepted at the 95% confidence level (*P* < 0.05). Animals were randomly allocated to each experimental group.

## Supplementary information

Supplemental file word

Supplementary Figure 1

Supplementary Figure 2

Supplementary Figure 3

Supplementary Figure 4

Supplementary Figure 5

## Data Availability

The data underlying this article will be shared on reasonable request to the corresponding author.

## References

[1] Ferriero DM (2004). Neonatal brain injury. N. Engl J Med.

[2] Perlman JM (2004). Brain injury in the term infant. Semin Perinatol.

[3] Volpe JJ (1992). Brain injury in the premature infant-current concepts of pathogenesis and prevention. Biol Neonate.

[4] Vannucci RC (2000). Hypoxic-ischemic encephalopathy. Am J Perinatol.

[5] Vannucci SJ, Hagberg H (2004). Hypoxia-ischemia in the immature brain. J Exp Biol.

[6] Walsh BH, Murray DM, Boylan GB (2011). The use of conventional EEG for the assessment of hypoxic ischaemic encephalopathy in the newborn. Clin Neurophysiol.

[7] Kirino T (2002). Ischemic tolerance. J Cereb Blood Flow Metab J Cereb Blood Flow Metab.

[8] Dirnagl U, Simon RP, Hallenbeck JM (2003). Ischemic tolerance and endogenous neuroprotection. Trends Neurosci.

[9] Gidday JM (2006). Cerebral preconditioning and ischaemic tolerance. Nat Rev Neurosci.

[10] Sart S, Ma T, Li Y (2014). Preconditioning stem cells for in vivo delivery. Biores Open Access.

[11] Ara J, De Montpellier S (2013). Hypoxic-preconditioning enhances the regenerative capacity of neural stem/progenitors in subventricular zone of newborn piglet brain. Stem Cell Res.

[12] Silva-Vargas V, Crouch EE, Doetsch F (2013). Adult neural stem cells and their niche: a dynamic duo during homeostasis, regeneration, and aging. Curr Opin Neurobiol.

[13] Hevner RF, Hodge RD, Daza RA, Englund C (2006). Transcription factors in glutamatergic neurogenesis: conserved programs in neocortex, cerebellum, and adult hippocampus. Neurosci Res.

[14] Pataskar A, Jung J, Smialowski P, Noack F, Calegari F, Straub T (2016). NeuroD1 reprograms chromatin and transcription factor landscapes to induce the neuronal program. EMBO J.

[15] Aprea J, Nonaka-Kinoshita M, Calegari F (2014). Generation and characterization of Neurod1-CreER(T2) mouse lines for the study of embryonic and adult neurogenesis. Genesis.

[16] Guillemot F (2007). Cell fate specification in the mammalian telencephalon. Prog Neurobiol.

[17] Gao Z, Ure K, Ables JL, Lagace DC, Nave K-A, Goebbels S (2009). Neurod1 is essential for the survival and maturation of adult-born neurons. Nat Neurosci.

[18] Tozuka Y, Fukuda S, Namba T, Seki T, Hisatsune T (2005). GABAergic excitation promotes neuronal differentiation in adult hippocampal progenitor cells. Neuron.

[19] Toth AB, Shum AK, Prakriya M (2016). Regulation of neurogenesis by calcium signaling. Cell Calcium.

[20] Pignataro G, Cuomo O, Vinciguerra A, Sirabella R, Esposito E, Boscia F (2013). NCX as a key player in the neuroprotection exerted by ischemic preconditioning and postconditioning. Adv Exp Med Biol.

[21] Cerullo P, Brancaccio P, Anzilotti S, Vinciguerra A, Cuomo O, Fiorino F (2018). Acute and long-term NCX activation reduces brain injury and restores behavioral functions in mice subjected to neonatal brain ischemia. Neuropharmacology.

[22] Fukuda S, Kato F, Tozuka Y, Yamaguchi M, Miyamoto Y, Hisatsune T (2003). Two distinct subpopulations of nestin-positive cells in adult mouse dentate gyrus. J Neurosci.

[23] Wiese C, Rolletschek A, Kania G, Blyszczuk P, Tarasov KV, Tarasova Y (2004). Nestin expression: a property of multilineage progenitor cells?. Cell Mol Life Sci.

[24] Berry SE, Andruszkiewicz P, Chun JL, Hong J (2013). Nestin expression in end-stage disease in dystrophin-deficient heart: implications for regeneration from endogenous cardiac stem cells. Stem Cells Transl Med.

[25] UnalCevik I, Dalkara T (2003). Intravenously administered propidium iodide labels necrotic cells in the intact mouse brain after injury. Cell Death Differ.

[26] Carloni S, Carnevali A, Cimino M, Balduini W (2007). Extended role of necrotic cell death after hypoxia-ischemia-induced neurodegeneration in the neonatal rat. Neurobiol Dis.

[27] Verslegers M, Van HoveI I, Buyens T, Dekeyster E, Knevels E, Moons L (2013). Identification of MMP-2 as a novel enhancer of cerebellar granule cell proliferation. Mol Cell Neurosci.

[28] Furutachi S, Matsumoto A, Nakayama KI, Gotoh Y (2013). p57 controls adult neural stem cell quiescence and modulates the pace of lifelong neurogenesis. EMBO J.

[29] Gidday JM, Fitzgibbons JC, Shah AR, Park TS (1994). Neuroprotection from ischemic brain injury by hypoxic preconditioning in the neonatal rat. Neurosci Lett.

[30] Vinciguerra A, Cuomo O, Cepparulo P, Anzilotti S, Brancaccio P, Sirabella R (2018). Models and methods for conditioning the ischemic brain. J Neurosci Methods.

[31] Bossenmeyer C, Chihab R, Muller S, Schroeder H, Daval JL (1998). Hypoxia/reoxygenation induces apoptosis through biphasic induction of protein synthesis in cultured rat brain neurons. Brain Res Mol Brain Res.

[32] Bossenmeyer-Pourié C, Lièvre V, Grojean S, Koziel V, Pillot T, Daval JL (2002). Sequential expression patterns of apoptosis- and cell cycle-related proteins in neuronal response to severe or mild transient hypoxia. Neuroscience.

[33] Kokaia Z, Lindvall O (2003). Neurogenesis after ischaemic brain insults. CurrOpinNeurobiol.

[34] Pourie G, Blaise S, Trabalon M, Nédélec E, Guéant J-L, Daval J-L (2006). Mild, non-lesioning transient hypoxia in the newborn rat induces delayed brain neurogenesis associated with improved memory scores. Neuroscience.

[35] Yin W, Signore AP, Iwai M, Cao G, Gao Y, Johnnides MJ (2007). Preconditioning suppresses inflammation in neonatal hypoxic ischemia via Akt activation. Stroke.

[36] Romijn HJ, Hofman MA, Gramsbergen A (1991). At what age is the developing cerebral cortex of the rat comparable to that of the full-term newborn human baby?. Early Hum Dev.

[37] Pressler R, Auvin S (2013). Comparison of brain maturation among species: an example in translational research suggesting the possible use of bumetanide in newborn. Front Neurol.

[38] Nicola Z, Fabel K, Kempermann G (2015). Development of the adult neurogenic niche in the hippocampus of mice. Front Neuroanat.

[39] Pignataro G, Sirabella R, Anzilotti S, Di Renzo G, Annunziato L. Does Na^+^/Ca^2+^ exchanger, NCX, represent a new druggable target in stroke intervention? Transl Stroke Res. 2014;5:145–55. 10.1007/s12975-013-0308-8. 10.1007/s12975-013-0308-8 24323727

[40] Secondo A, Pignataro G, Ambrosino P, Pannaccione A, Molinaro P, Boscia F (2015). Pharmacological characterization of the newly synthesized 5-amino-N-butyl-2-(4-ethoxyphenoxy)-benzamide hydrochloride (BED) as a potent NCX3 inhibitor that worsens anoxic injury in cortical neurons, organotypic hippocampal cultures, and ischemic brain. ACS Chem Neurosci.

[41] Secondo A, Esposito A, Sirabella R, Boscia F, Pannaccione A, Molinaro P (2015). Involvement of the Na+/Ca2+ exchanger isoform 1 (NCX1) in neuronal growth factor (NGF)-induced neuronal differentiation through Ca2+-dependent Akt phosphorylation. J Biol Chem.

[42] Pignataro G, Boscia F, Esposito E, Sirabella R, Cuomo O, Vinciguerra A (2012). NCX1 and NCX3: two new effectors of delayed preconditioning in brain ischemia. Neurobiol Dis.

[43] Sisalli MJ, Secondo A, Esposito A, Valsecchi V, Savoia C, Di Renzo GF (2014). Endoplasmic reticulum refilling and mitochondrial calcium extrusion promoted in neurons by NCX1 and NCX3 in ischemic preconditioning are determinant for neuroprotection. Cell Death Differ.

[44] Sokolow S, Manto M, Gailly P, Molgó J, Vandebrouck C, Vanderwinden J-M (2004). Impaired neuromuscular transmission and skeletal muscle fiber necrosis in mice lacking Na/Ca exchanger 3. J Clin Invest.

[45] Molinaro P, Cuomo O, Pignataro G, Boscia F, Sirabella R, Pannaccione A (2008). Targeted disruption of Na+/Ca2+ exchanger 3 (NCX3) gene leads to a worsening of ischemic brain damage. J Neurosci.

[46] Kilkenny C, Browne WJ, Cuthill IC, Emerson M, Altman DG (2010). Improving bioscience research reporting: the ARRIVE guidelines for reporting animal research. PLoS Biol.

[47] Rice JE, Vannucci RC, Brierley JB (1981). The influence of immaturity on hypoxic-ischemic brain damage in the rat. Ann Neurol.

[48] Vannucci RC, Towfighi J, Vannucci SJ (2004). Secondary energy failure after cerebral hypoxia-ischemia in the immature rat. J Cereb Blood Flow Metab.

[49] Valsecchi V, Anzilotti S, Serani A, Laudati G, Brancaccio P, Guida N (2020). miR-206 reduces the severity of motor neuron degeneration in the facial nuclei of the brainstem in a mouse model of SMA. Mol Ther.

[50] Bederson JB, Pitts LH, Tsuji M, Nishimura MC, Davis RL, Bartkowskiet H (1986). Rat middle cerebral artery occlusion: evaluation of the model and development of a neurologic examination. Stroke.

[51] Anzilotti S, Tornincasa M, Gerlini R, Conte A, Brancaccio P, Cuomo O (2015). Genetic ablation of homeodomain-interacting protein kinase 2 selectively induces apoptosis of cerebellar Purkinje cells during adulthood and generates an ataxic-like phenotype. Cell Death Dis.

[52] Shigemoto-Mogami Y, Hoshikawa K, Goldman JE, Sekino Y, Sato K (2014). Microglia enhance neurogenesis and oligodendrogenesis in the early postnatal subventricular zone. J Neurosci.

[53] Cuomo O, Cepparulo P, Anzilotti S, Serani A, Sirabella R, Brancaccio P (2019). Anti-miR-223-5p ameliorates ischemic damage and improves neurological function by preventing NCKX2 downregulation after ischemia in rats. Mol Ther Nucleic Acids.

[54] Anzilotti S, Brancaccio P, Simeone G, Valsecchi V, Vinciguerra A, Secondo A (2018). Preconditioning, induced by sub-toxic dose of the neurotoxin L-BMAA, delays ALS progression in mice and prevents Na +/Ca 2+ exchanger 3 downregulation. Cell Death Dis.

[55] Anzilotti S, Valsecchi V, Brancaccio P, Guida N, Laudati G, Tedeschi V (2021). Prolonged NCX activation prevents SOD1 accumulation, reduces neuroinflammation, ameliorates motor behavior and prolongs survival in a ALS mouse model. Neurobiol Dis.

[56] Sanguigno L, Casamassa A, Funel N, Minale M, Riccio R, Riccio S (2018). Triticum vulgare extract exerts an anti-inflammatory action in two in vitro models of inflammation in microglial cells. PLoS ONE.

[57] Paxinos G, Watson C, Kassem M, Halliday G. Atlas of the developing mouse brain 2020. 2nd edn. San Diego, California, USA: Academic Press, Elsevier, 2020.

[58] Gustorff C, Scheuer T, Schmitz T, Schmitz T, Bührer C, Endesfelde S (2021). GABA B receptor-mediated impairment of intermediate progenitor maturation during postnatal hippocampal neurogenesis of newborn rats. Front Cell Neurosci.

[59] Ten VS, Bradley-Moore M, Gingrich JA, Stark RI, Pinsky DJ (2003). Brain injury and neurofunctional deficit in neonatal mice with hypoxic-ischemic encephalopathy. Behav Brain Res.

[60] Fan LW, Tien LT, Zheng B, Pang Y, Rhodes PG, Cai Z (2010). Interleukin-1beta-induced brain injury and neurobehavioral dysfunctions in juvenile rats can be attenuated by alpha-phenyl-n-tert-butyl-nitrone. Neuroscience.

