# Supplementary material for: Preconditioning in hypoxic-ischemic neonate mice triggers Na+-Ca2+ exchanger-dependent neurogenesis
Source: Cell Death Discov. 2022 Jul 13;8:318. doi: 10.1038/s41420-022-01089-z (PMC9279453; doi:10.1038/s41420-022-01089-z)
Supplement: Supplementary file 1 — Supplemental file word [file 41420_2022_1089_MOESM1_ESM.docx]

**SUPPLEMENTAL MATERIAL**

**MATERIAL AND METHODS**

**Muscle analysis**

Gastrocnemius muscle analysis were performed as previously described^35^. Sections were stained with a mouse monoclonal anti-Nestin (1:200); anti-alpha Tubulin 4a (1:400); nuclei were counterstained with Hoechst~~.~~

**Cell-counting analysis**

The number of BrdU and PSA-NCAM positive cells was determined in matched sections (40 µm thickness) of SVZ of p11 C57BL/6 mice, by manual counting at ×40 magnification. Three selected rostro-caudal sections were obtained from the region 3.27 mm to 3.63 mm from rostral cortex^38^ and included in the analysis. The average value of all the sections of each animal was determined.

**LEGENDS OF SUPPLEMENTARY FIGURES**

**FIGURE S1:**

**Nestin expression in different tissues of wild-type mice.**

Representative confocal images of gastrocnemius muscle and dentate gyrus of hippocampus respectively of adult (P90) and pup mice (P11), subjected to PC+HI. Single staining of Nestin (A, B), α-tubulin (C, D), Hoechst (E, F), and merge (G, H). Scale bar 25μm. Arrows indicate Nestin expressing cells.

**FIGURE S2:**

**Effect of Preconditioning stimulus on neuroblasts in SVZ of HI mice.**

Representative confocal images of SVZ of control, HI, and PC+HI groups. Single staining of BrdU (A, B, C), PSA-NCAM (D, E, F), Hoechst (G, H, I), and merge (J, K, L). Scale bar 75 μm. Arrows indicate PSA-NCAM and BrdU/PSA-NCAM expressing cells. (B) Cell counting analysis of neuroblasts expressed as the total number of PSA-NCAM^+^ cells in SVZ of P11 mice. (C) Cell counting analysis of proliferating neuroblasts expressed as the total number of BrdU/PSA-NCAM^+^ cells in SVZ of P11 mice. (B-C) Data are expressed as mean ± SD (n=5). *P<0.05 versus control mice. #P<0.05 versus HI mice. P values were obtained by using 1-way ANOVA with Newman Keuls correction for multiple comparisons.

**FIGURE S3:**

**Nestin expression in the hippocampal dentate gyrus of P11 mice**.

Representative confocal images of dentate gyrus of control, PC, HI, and PC+HI groups. Single staining of Nestin (A, D, G, J); Hoechst (B, E, H, K), and merge (C, F, I, L) in mouse dentate gyrus. Scale bar 25μm. Arrows indicate Nestin expressing cells.

**FIGURE S4:**

**Contribution of NCX3 on neuroprotection mediated by HPC.**

(A) Cresyl violet stained representative rostral-caudal brain sections of preconditioned wild-type and preconditioned ncx3-/- mice are included on top of the figure. Brain damage induced in mice was evaluated as % of ischemic damage at postnatal day eleven. Data are expressed as mean ± SD (n=3-4). (B) Representative confocal images of NeuroD1 staining in DG of preconditioned (wild-type) and preconditioned (ncx3-/-) mice. Single staining of NeuroD1 (A, D), Hoechst (B, E), and merge (C, F). Scale bar 75 μm. Arrows indicate NeuroD1 expressing cells.

**FIGURE S5:**

**NeuroD1 staining in dentate gyrus of mice subjected to hypoxic-ischemic insult.**

Representative confocal images of dentate gyrus of hypoxic-ischemic wild-type and ncx3-/- groups. On the left are reported two representative brain section stained with cresyl violet. Single staining of NeuroD1 (A, B), Hoechst (C, D), and merge (E, F). Scale bar 75 μm. Arrows indicate NeuroD1 expressing cells.
