# Supplementary figures and images for: Preconditioning in hypoxic-ischemic neonate mice triggers Na+-Ca2+ exchanger-dependent neurogenesis
Source: Cell Death Discov. 2022 Jul 13;8:318. doi: 10.1038/s41420-022-01089-z (PMC9279453; doi:10.1038/s41420-022-01089-z)

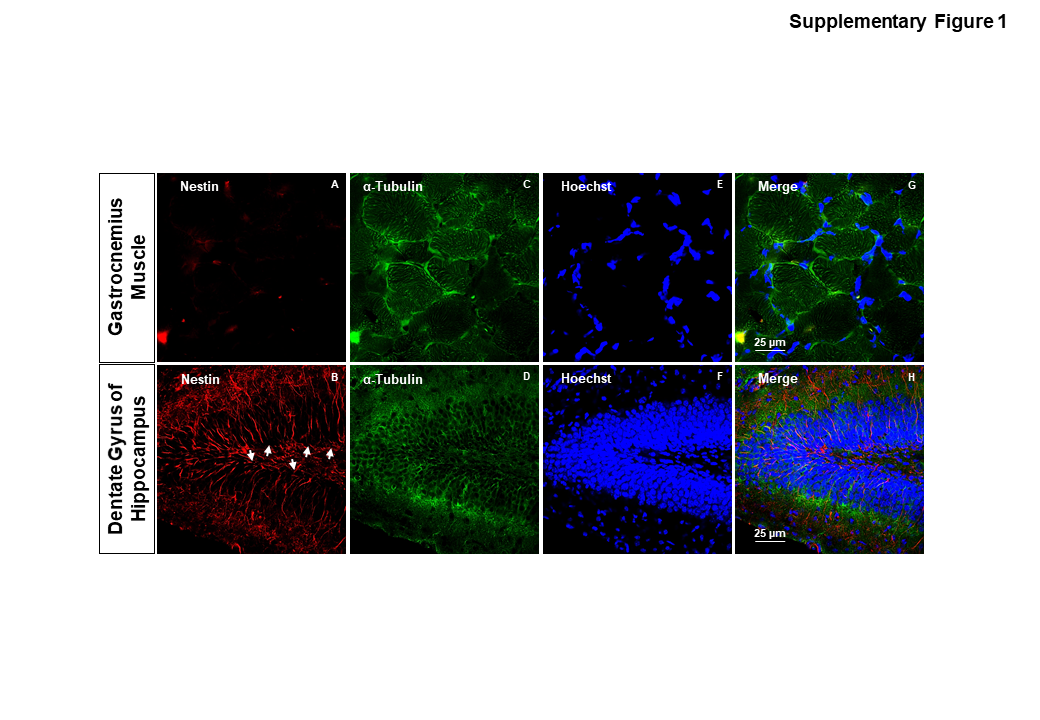

Supplement: Supplementary file 2 — Supplementary Figure 1 [file 41420_2022_1089_MOESM2_ESM.tif]

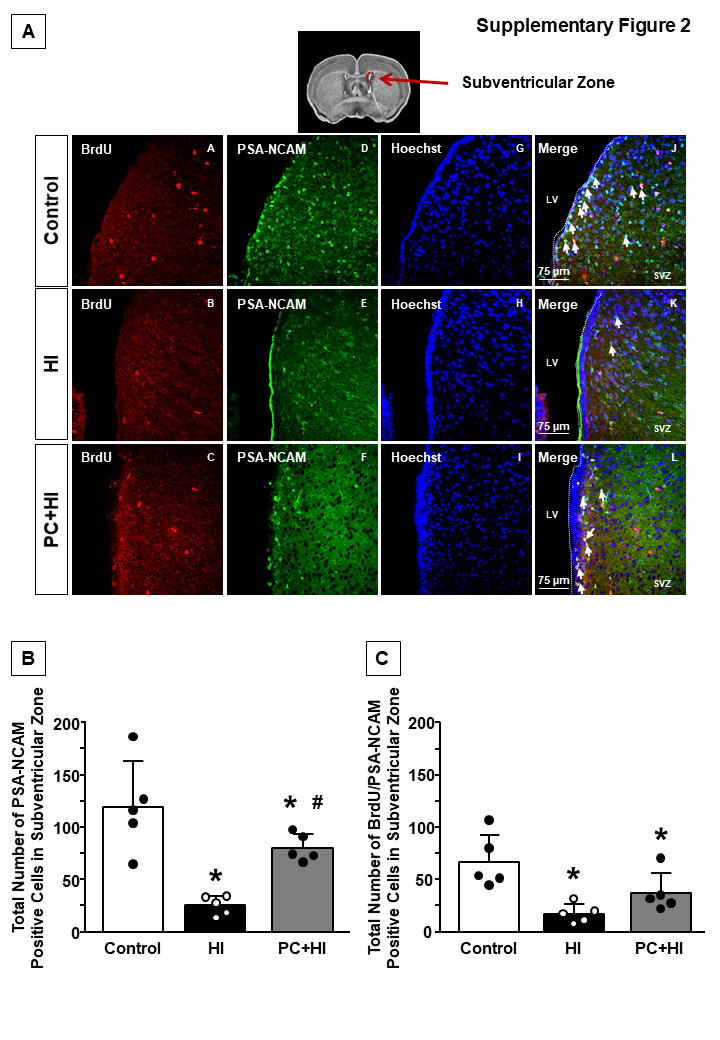

Supplement: Supplementary file 3 — Supplementary Figure 2 [file 41420_2022_1089_MOESM3_ESM.tif]

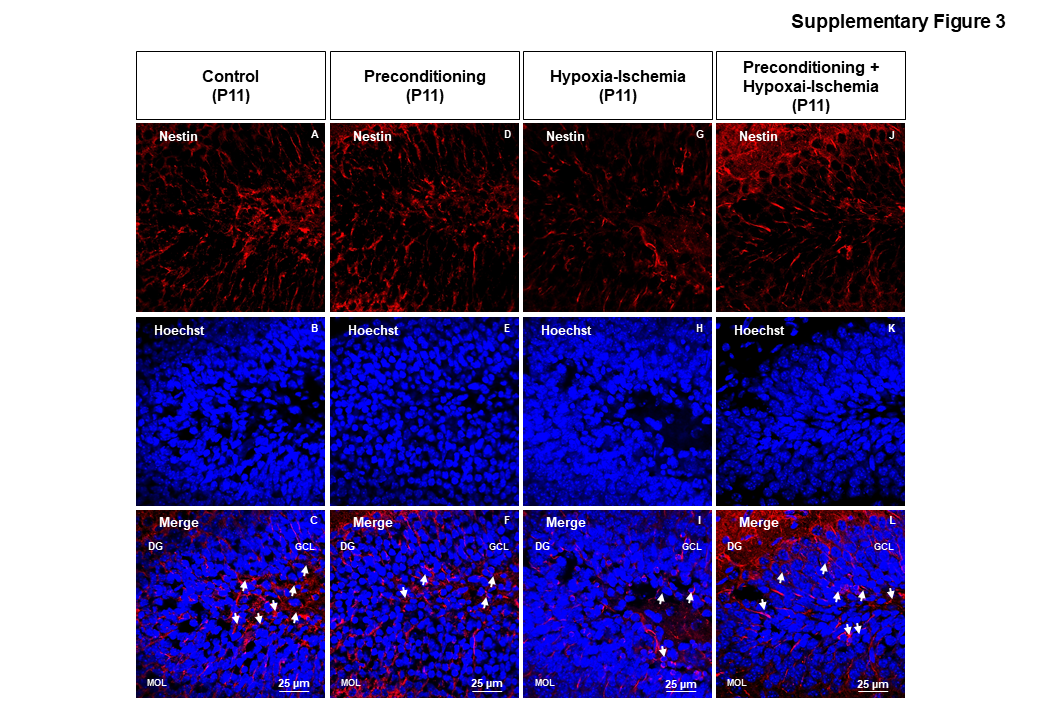

Supplement: Supplementary file 4 — Supplementary Figure 3 [file 41420_2022_1089_MOESM4_ESM.tif]

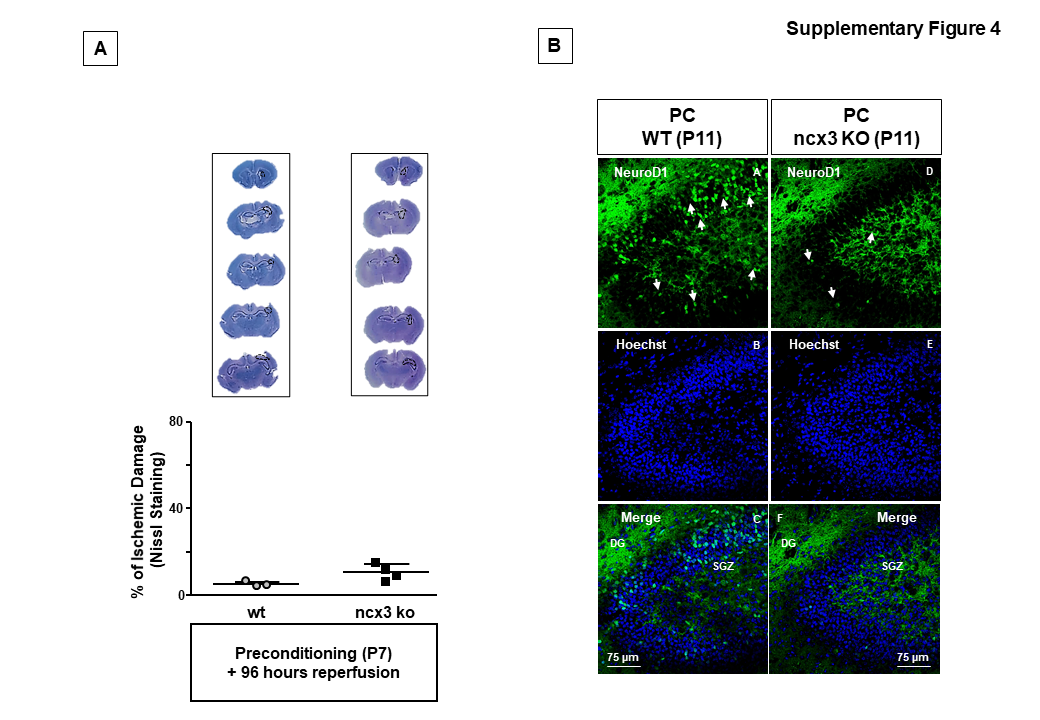

Supplement: Supplementary file 5 — Supplementary Figure 4 [file 41420_2022_1089_MOESM5_ESM.tif]

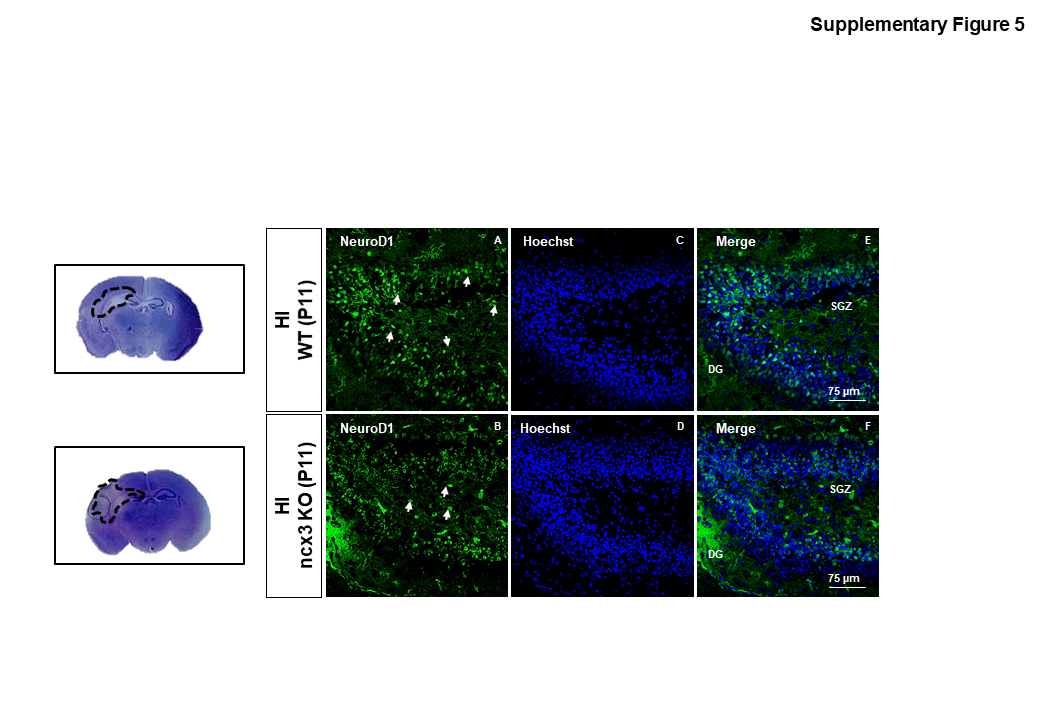

Supplement: Supplementary file 6 — Supplementary Figure 5 [file 41420_2022_1089_MOESM6_ESM.tif]
